# Changes in Retinal OCT and Their Correlations with Neurological Disability in Early ALS Patients, a Follow-Up Study

**DOI:** 10.3390/brainsci9120337

**Published:** 2019-11-24

**Authors:** Pilar Rojas, Rosa de Hoz, Ana I. Ramírez, Antonio Ferreras, Elena Salobrar-Garcia, José L. Muñoz-Blanco, José L. Urcelay-Segura, Juan J. Salazar, José M. Ramírez

**Affiliations:** 1 General University Hospital Gregorio Marañón, Ophthalmic Institute of Madrid, 28007 Madrid, Spain; pilar.rojas.lozano@gmail.com (P.R.); urzemendi@telefonica.net (J.L.U.-S.); 2 Ramón Castroviejo Ophthalmological Research Institute, Complutense University of Madrid, 28040 Madrid, Spain; rdehoz@med.ucm.es (R.d.H.); airamirez@med.ucm.es (A.I.R.); elenasalobrar@med.ucm.es (E.S.-G.); 3 Department of Immunology, Ophthalmology and Otorhinolaryngology, School of Optics and Optometry, Complutense University of Madrid, 28037 Madrid, Spain; 4 Miguel Servet University Hospital, Aragonese Institute of Health Sciences, 50009 Zaragoza, Spain; aferreras@msn.com; 5 Department of Immunology, Ophthalmology and Otorhinolaryngology, School of Medicine, Complutense University, 28040 Madrid, Spain; 6 Department of Neurology, ALS-Neuromuscular Unit, Gregorio Marañón Health Research Institute, 28007 Madrid, Spain; joseluis.munoz@madrid.org

**Keywords:** amyotrophic lateral sclerosis, ALS, OCT, retina, Optic nerve, eye, neurodegenerative

## Abstract

Background: To compare early visual changes in amyotrophic lateral sclerosis (ALS) patients with healthy controls in a baseline exploration, to follow-up the patients after 6 months, and to correlate these visual changes with neurological disability. Methods: All patients underwent a comprehensive neurological and ophthalmological examination. A linear mixed analysis and Bonferroni *p*-value correction were performed, testing four comparisons as follows: Control baseline vs. control follow-up, control baseline vs. ALS baseline, control follow-up vs. ALS follow-up, and ALS baseline vs. ALS follow-up. Results: The mean time from the diagnosis was 10.80 ± 5.5 months. The analysis of the optical coherence tomography (OCT) showed: (1) In ALS baseline vs. control baseline, a macular significantly increased thickness of the inner macular ring temporal and inferior areas; (2) in ALS follow-up vs. ALS baseline, a significant macular thinning in the inner and outer macular ring inferior areas; (3) in ALS follow-up vs. ALS baseline, a significant peripapillary retinal nerve fiber layer (pRNFL) thinning in the superior and inferior quadrants; and (4) ALS patients showed a moderate correlation between some OCT pRNFL parameters and Amyotrophic Lateral Sclerosis Functional Rating Scale-Revised (ALSFRS-R) score. Conclusion: The OCT showed retinal changes in patients with motoneuron disease and could serve as a complementary tool for studying ALS.

## 1. Introduction

Amyotrophic lateral sclerosis (ALS) is the most common form of progressive motor neuron disease. Worldwide, the incidence of ALS ranges from 0.3 to 2.5 cases per year per 100,000 people [1]. It is more common in men than in women, with a ratio of 1.5:1 [1]. ALS is a neurodegenerative disease characterized by rapid progression, which affects first and second motor neurons to different degrees, causing muscle fiber atrophy. Patients with ALS die within two to three years from diagnosis, usually due to respiratory failure [1,2]. Classically, eye motor nuclei were thought to be unaffected. However, some studies have found abnormal ocular movements in these patients [3,4,5]. No single specific test exists for ALS diagnosis; it is a diagnosis of exclusion based on the initial symptoms, the progression of the disease, and tests to eliminate overlapping conditions. Finally, the degree of neurological disability is determined by the Amyotrophic Lateral Sclerosis Functional Rating Scale-Revised (ALSFRS-R) [6].

To date, many studies have focused on assessing the possible participation of non-motor areas of the central nervous system (CNS) in ALS [7,8,9,10,11]. These patients have demonstrated [7]:

(1) An overall reduction in brain volume [10,11], with a loss of focal grey matter and changes in white matter in non-motor areas [9,12];
(2) Cognitive impairment [8];
(3) Small fiber neuropathy [13];
(4) Abnormal evoked potentials [14,15,16,17];
(5) Oculomotor dysfunction [3,4,18,19,20]; and
(6) Astrogliosis in non-motor areas, specifically in the occipital area [21].

Optical coherence tomography (OCT) is a non-invasive imaging technique that is accurate, objective, and reproducible. OCT can provide high-resolution images of retinal layers [22] and detect neuroaxonal retinal abnormalities in many neuroinflammatory and neurodegenerative diseases [23,24,25,26,27,28,29,30,31,32,33,34,35,36,37,38,39]. Some researchers have analyzed changes in the visual pathway (a non-motor neuron area) using OCT in ALS patients [7,40,41,42,43,44,45]. These studies are not homogeneous because they included not only different clinical symptoms of ALS (bulbar, frontotemporal dementia, or spinal onset), but also different stages of disease progression. Therefore, the results, in many cases, are contradictory. All these works are cross-sectional without patient follow-up. Longitudinal studies are needed to further investigate the temporal evolution of ALS in the retina and the optic nerve.

The aims of this study were to compare early visual changes in ALS patients with healthy controls in a baseline exploration, to follow up with the patients after 6 months, and to correlate these visual changes with neurological disability.

## 2. Material and Methods

### 2.1. Participants: Study Groups and Selection Criteria

The study protocol adhered to the tenets of the Helsinki Declaration and was approved by the Gregorio Marañón Hospital (HGUGM) Ethical Committee (NCT03285204). Each subject provided written informed consent before entering the study.

This prospective study was conducted at the HGUGM and Ramón Castroviejo Ophthalmologic Research Institute in the Complutense University of Madrid, Spain. Patients were diagnosed in the ALS-neuromuscular unit of the Neurology Department, based on El Escorial criteria [46]. We performed the ophthalmological examination only if patients met the following criteria: (1) Being diagnosed in the first 18 months after the onset of the motor symptoms, (2) having spinal onset, (3) being sporadic ALS, and (4) not having clinical cognitive impartment. Only 10 ALS patients fulfilled these criteria. Thus, we selected 20 eyes from the 10 ALS patients (ALS baseline group). We were only able to follow-up with 5 out of the 10 initial patients due to very poor health conditions or death by respiratory distress or intercurrent diseases. Therefore, we studied the 10 eyes of these 5 patients (ALS follow-up group). The control group was selected from the patients’ relatives, mainly the spouse, and volunteers. We studied 38 eyes from 19 healthy controls (control baseline group) and followed up with them after 6 months (control follow-up group). 

### 2.2. Study Protocol

A multidisciplinary team examined each patient. This team included neurologists, pulmonologists, and ophthalmologists. All patients underwent an exhaustive complete neurological examination, including the ALSFRS-R scale, with the same neurologist [6]. For ophthalmological examination, both eyes of each patient were analyzed. All participants, ALS and controls, met the following inclusion criteria: (1) Being free of ocular disease, (2) Evaluation of Aged-Related Eye Disease (AREDS) Clinical Lens Standards <2, (3) being free of systemic disorders affecting vision, (4) having a best corrected visual acuity (BCVA) of more than 20/40, (5) having less than ±5 sphere–cylindrical refractive error, and (6) having intraocular pressure (IOP) less than 20 mmHg. 

For screening, all ALS patients and control subjects underwent a complete ophthalmologic examination, including an assessment of BCVA, refraction, anterior segment biomicroscopy, measurement of intraocular pressure (IOP) with Goldman applanation tonometer (AT900, Haag-Streit, Köniz, Switzerland), dilated fundus examination, and a spectral-domain Cirrus HD-OCT Model 4000 (Carl Zeiss Meditec, Dublin, CA, USA; software version 6.2) analysis using Optic Disc 200 × 200 and Macular cube 512 × 128 scanning protocols [47,48]. Macular thickness (MT), ganglion cell complex (GCC), cube volume, average cube thickness, and peripapillary retinal nerve fiber layer (pRNFL) thickness were measured via OCT Cirrus after pupil dilation. The mean values were considered for statistical analysis. All tests were performed by the same ophthalmologist under the same conditions for the baseline evaluation and the follow-up (after 6 months). 

**Figure 1:**
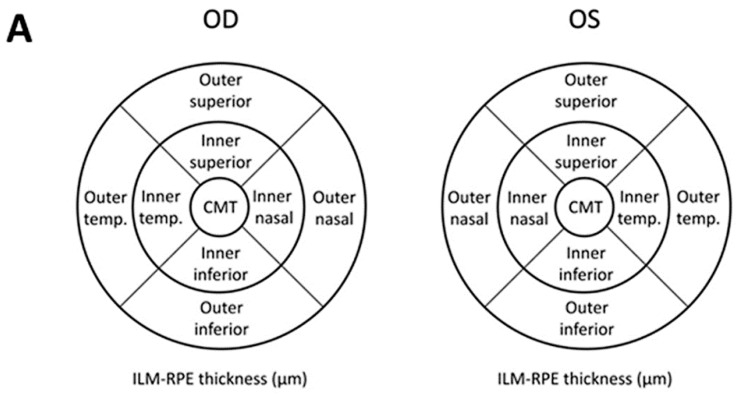

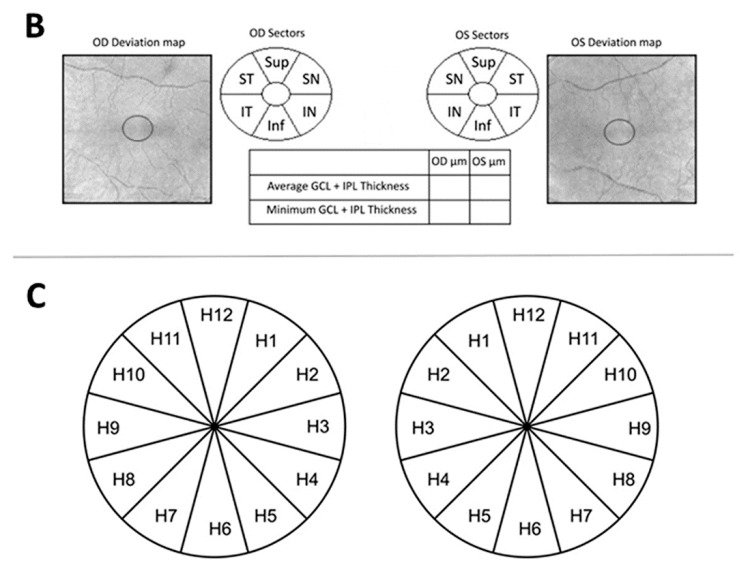
Optic coherence tomography (OCT) analysis of the retina: (**A**) Coding for the macular Early Treatment Diabetic Retinopathy Study (ETDRS) grid, (**B**) ganglion cell complex (GCC) parameters, and (**C**) peripapillary retinal nerve fiber layer (pRNFL) thicknesses. OD: Right eye; OI: Left eye; CMT: Central macular thickness; ILM: Inner limiting membrane; RPE: Retinal pigment epithelium; Sup: Superior; SN: Supero-nasal; IN: Infero-nasal; Inf: Inferior; IT: Infero-temporal; ST: Supero-temporal; GCL: Ganglion cell layer; IPL: Inner plexiform layer; µm: Microns; H: Clock-hour position.

As in the Early Treatment Diabetic Retinopathy Study (ETDRS) [47], MT data were displayed in three concentric rings centered in the fovea. These rings were distributed as follows: A central macular thickness (CMT) with a diameter of 1 mm, an inner macular ring (IMR) with a diameter of 3 mm, and an outer macular ring (OMR) with a diameter of 6 mm. The inner and outer rings were each divided into four quadrants (superior, inferior, nasal, and temporal). The total volume of the macula, as determined using OCT, was also used. The GCC covers two inner layers of the retina: IPL (inner plexiform layer) and GCL (ganglion cell layer), which were measured by special segmentation software provided by Cirrus HD-OCT Model 4000 (Carl Zeiss Meditec, Dublin, CA, USA; software version 6.2), checked by the ophthalmologist and re-centered if needed. The pRNFL average thickness was measured. Peripapillary RNFL was also segmented into four quadrants (superior, temporal, inferior, and nasal), and in 12 clock hours (H) (H3 denoting nasal, H6 inferior, H9 temporal, and H12 superior in a right eye). Left eyes (LE) were converted to right eyes (RE) with a specular image (Figure 1). The analyzed area was centered manually and the absence of segmentation errors was confirmed for each scan. A good scan was one that met the criterion of a signal-to-noise ratio (SNR) >7/10. All measurements are provided in microns (µm), according to the calibration provided by the manufacturers, and the total volume in cubic millimeters (mm^3^).

Visual fields (VFs) were analyzed using a Humphrey Field Analyzer 750i (Humphrey Zeiss Systems, Dublin, CA, USA; 24-2) with the Swedish interactive threshold algorithm (SITA) Fast 24-2 strategy. Mean deviation (MD), pattern standard deviation (PSD), and visual field index (VFI) were used as perimetric indices of generalized sensitivity loss. Localized scotomas and percentages of useful residual vision of the patient were studied. A scotoma was defined as a cluster of at least three points with lower sensitivity than normal values (including two or more points with a *p*-value of less than 0.5%) on the pattern deviation probability map of the Humphrey Field Analyzer 750i (Humphrey Zeiss Systems, Dublin, CA, USA; 24-2) 24-2 SITA-Fast program. Points with diminished sensitivity adjacent to the blind spot were not considered parts of scotomas [49].

### 2.3. Statistical Analysis

Data for the statistical analysis were introduced and processed in SPSS 23.0 (SPSS Inc.©, IBM Corporation, Somers, NY, USA) and StataCorp. 2015 (Stata Statistical Software: Release 14. College Station, TX: StataCorp LLC, College Station, TX, USA).

Minimum sample size for each group was estimated in seven eyes (ratio of sample sizes in the control group/ALS group = 1) on the basis of a difference in the average pRNFL thickness of 25.1 µm and standard deviations (SDs) of 10.3 and 10.9, a type 1 error rate of 0.05, and a power of 95% (MedCalc software). Difference of pRNFL thickness based on Ferreras et al.’s study [50].

The normality of distribution was assessed with the Kolmogorov–Smirnov test. Non-parametric tests were used because data were not normally distributed. A mixed linear analysis was used, both in the baseline and the follow-up analysis, in order not to overestimate the statistical power of the results because both eyes of the patients were considered for the study. For the longitudinal part, we only compared the patients who attended the second visit with their baseline exploration. We performed four comparisons as follows: Control baseline vs. control follow-up, control baseline vs. ALS baseline, control follow-up vs. ALS follow-up, and ALS baseline vs. ALS follow-up. To verify any asymmetries between right eyes (RE) and left eyes (LE) in the OCT, a Wilcoxon test was also performed. To correlate functional and anatomical values with ALSFR-R, a Spearman rho test was employed.

Data were reported as mean values ± SD. All *p*-values were corrected using Bonferroni correction. A *p*-value < 0.05 was considered statistically significant.

## 3. Results

Demographic and clinical data of ALS patients and control groups are shown in Table 1. The duration of the illness from the diagnosis to the ophthalmological evaluation ranged from 1 to 18 months (10.80 ± 5.5 months). All patients had a fast-progressive disease; therefore, half of them could not attend the second exploration due to poor health condition or death. 

### 3.1. ALSFRS-R

The ALSFRS-R score showed statistically significant differences in the comparisons between control baseline (48.00 ± 0.00) vs. ALS baseline (29.50 ± 14.89) and control follow-up (48.00 ± 0.00) vs. ALS follow-up (35.6 ± 14.08) (Table 1).

### 3.2. Best-Corrected Visual Acuity (BCVA)

No significant differences were found in BCVA in the Snellen chart in any of the comparisons between groups (Table 1). 

### 3.3. Visual Field (VF)

Overall, in VF parameters, we did not find statistically significant differences. We only found a statistical difference in the percentage of fixation losses (% FL) in the comparison of ALS baseline vs. ALS follow-up (Table 1). 

### 3.4. Optical Coherence Tomography (OCT)

The comparison between control baseline vs. control follow-up did not show statistical differences in the OCT parameters (MT, GCC, pRNFL). 

### 3.5. Macular Thickness (MT)

**Figure 2:**
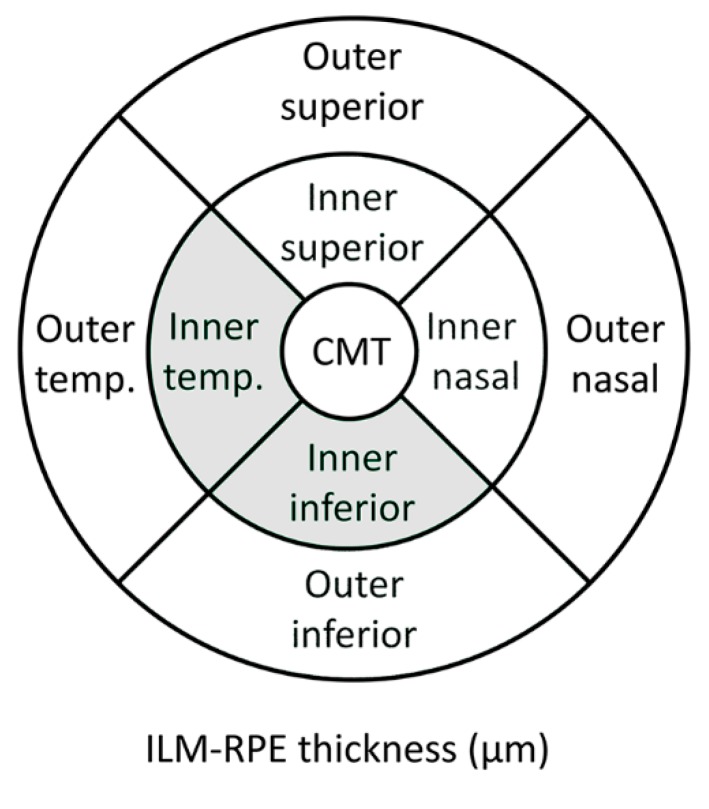
Scheme of the significant thickness changes between amyotrophic lateral sclerosis (ALS) baseline patients and control baseline group measured using OCT in a right eye. Grey denotes an increased thickness of the macular areas in ALS patients in the baseline exploration. CMT: Central macular thickness. Temp.: Temporal.

**Figure 3:**
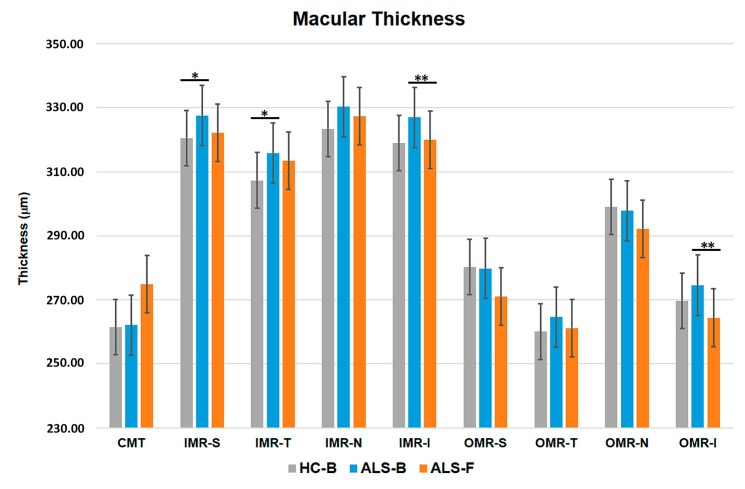
Macular thickness. Control baseline (*n* = 38) vs. ALS baseline (*n* = 20): A statistically significant increased thickness of temporal and inferior areas of the inner macular ring in ALS baseline patients. ALS baseline (*n* = 20) vs. ALS follow-up (*n* = 10): A statistically significant thinning of inferior areas of the inner and outer macular ring in ALS follow-up patients. HC: Healthy control; ALS: Amyotrophic lateral sclerosis, B: Baseline, F: Follow-up, OCT: Optic coherence tomography; CMT: Central macular thickness; IMR: Inner macular ring; OMR: Outer macular ring; S: Superior, T: Temporal, N: Nasal, I: Inferior. * *p* < 0.05 after Bonferroni correction in the comparison between control baseline vs. ALS baseline. ** *p* < 0.05 after Bonferroni correction in the comparison between ALS baseline vs. ALS follow-up.

The ALS baseline group showed a significant increased thickness in the inferior (326.91 ± 15.34) and temporal (315.85 ± 15.34) areas of IMR compared with the control baseline group (inferior = 319.00 ± 12.54; temporal = 307.32 ± 12.49) (*p* < 0.05; both instances). The comparison between ALS baseline vs. ALS follow-up revealed a significant thinning in the inferior areas of IMR (ALS baseline = 326.91 ± 15.34 and ALS follow-up = 320.00 ± 11.88) and OMR (ALS baseline = 274.42 ± 14.20 and ALS follow-up = 264.40 ± 16.34). Non-significant differences were observed in the comparison between control follow-up vs. ALS follow-up (Figure 2 and Figure 3; Table 2).

### 3.6. Ganglion Cell Complex (GCC)

Non-significant changes were found in the GCC thickness in any of the comparisons analyzed in the study (Table 3).

### 3.7. Peripapillary Retinal Nerve Fiber Layer (pRNFL)

**Figure 4:**
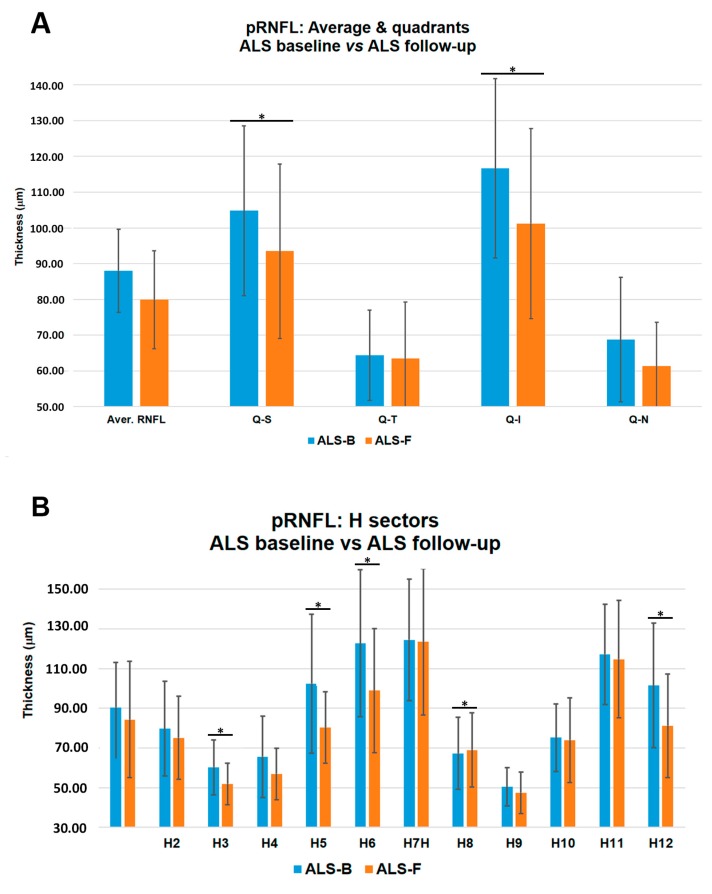
Retinal nerve fiber layer. (**A**) pRNFL, average and quadrants, ALS baseline (*n* = 20) vs. ALS follow-up (*n* = 10): A statistically significant thinning of superior and inferior quadrants in ALS follow-up patients. (**B**) pRNFL, H sectors, ALS baseline vs. ALS follow-up: A statistically significant thinning of sectors H3, H5, H6, and H12 and a statistically significant increased thickness of H8 in ALS follow-up patients are shown. * *p* < 0.05 after Bonferroni correction in the comparison between ALS baseline vs. ALS follow-up.

The comparison between ALS baseline vs. ALS follow-up showed significant thinning in the inferior (ALS baseline = 116.65 ± 25.08 and ALS follow-up = 101.20 ± 26.62) and superior (ALS baseline = 104.80 ± 23.80 and ALS follow-up = 93.50 ± 24.38) quadrants, and in sectors H3 (ALS baseline = 60.30 ± 13.82 and ALS follow-up = 51.90 ± 10.40), H5 (ALS baseline = 102.40 ± 35.00 and ALS follow-up = 80.30 ± 17.93), H6 (ALS baseline = 122.75 ± 37.06 and ALS follow-up = 99.00 ± 31.24), and H12 (ALS baseline = 101.50 ± 31.37 and ALS follow-up = 81.20 ± 26.15); and an increased thickness in sector H8 (ALS baseline = 67.35 ± 18.23 and ALS follow-up = 69.00 ± 18.74) (*p* < 0.05 in all instances, except for H6, *p* < 0.001; Table 4 and Figure 4).

### 3.8. Interocular Asymmetry

Overall, we found significant interocular asymmetries between LE and RE in:

(1) The ALS baseline group in the inferior-nasal area of GCC (RE: 80.60 ± 7.01 and LE: 73.80 ± 13.93 µm, *p* = 0.042) and in the sectors H7 (RE: 133.00 ± 24.69 and LE: 116.00 ± 34.62 µm, *p* = 0.022) and H9 (RE: 53.90 ± 11.51 and LE: 47.00 ± 5.93 µm, *p* = 0.020).
(2) The ALS follow-up group: In the supero-nasal quadrant of the GCC (RE: 79.00 ± 9.27 and LE: 76.00 ± 11.53 µm, *p* = 0.043), in the temporal quadrant of pRNFL (RE: 68.40 ± 16.04 and LE: 58.60 ± 15.66 µm, *p* = 0.039), and in sectors H8 (RE: 75.00 ± 18.55 and LE: 63.00 ± 18.87 µm, *p* = 0.043) and H9 (RE: 50.80 ± 12.40 and LE: 44.20 ± 8.11 µm, *p* = 0.043).

### 3.9. Spearman Correlations between OCT Measurements and ASLFRS-R Score

**Figure 5:**
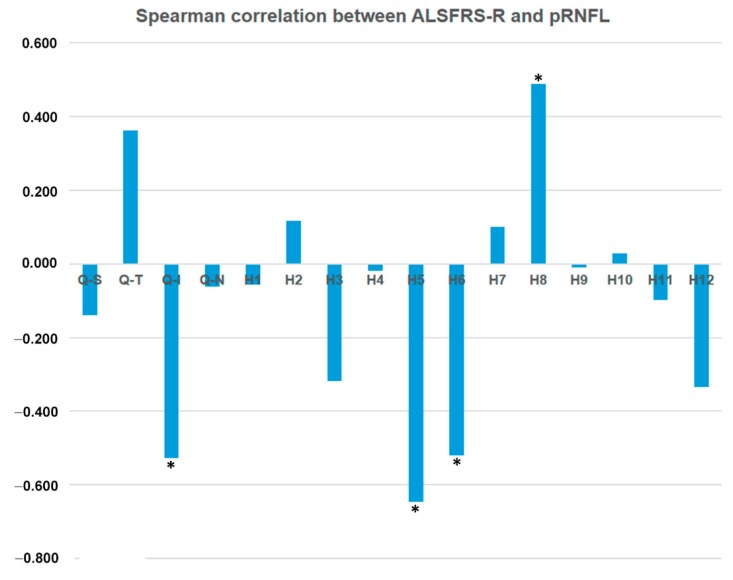
Spearman correlation between ALSFRS-R and pRNFL. Q: Quadrant; S: Superior; T: Temporal; N: Nasal, H: Hour. * *p* < 0.05.

In ALS baseline, neither functional (BCVA and VF) nor macular OCT parameters (MT, GCC) correlated with the ALSFRS-R score. However, some pRNFL OCT parameters correlated with ALSFRS-R (*p* < 0.05) in the inferior quadrant (*ρ* = −0.527) and sectors H5 (*ρ* = −0.647), H6 (*ρ* = −0.521), and H8 (*ρ* = +0.488) (Table 1, Table 2, Table 3 and Table 4 and Figure 5).

## 4. Discussion

ALS is considered a complex neurodegenerative disease, comprising motor and extra-motor symptoms such as cognitive impairment, behavioral deficits, abnormalities in both brainstem auditory and visual evoked potentials, and subtle visual defects [8,15,16,40,51]. Few studies, especially those using the OCT to determine visual anomalies, relate ALS with the visual system [7,16,40,41,42,43,44,45,52,53,54,55] because patients do not complain of visual problems, which are overshadowed by motor and respiratory symptoms. In this study, BCVA, VFs, and OCT were analyzed in patients with sporadic ALS and a spinal onset. The analyses were performed in a baseline study (*n* = 20) and in a 6-month follow-up, where only data of patients who were able to attend the second examination were considered (*n* = 10).

The degree of neurological disability was determined using ALSFRS-R [6]. In the ALS group (baseline and follow-up), ALSFRS-R was significantly decreased compared with the control group (baseline and follow-up). However, in the comparison between ALS baseline vs. ALS follow-up, the ALSFRS-R showed a false improvement, because five patients with worse ALSFRS-R scores could not attend the second exploration due to poor health conditions or death. 

In ALS patients (baseline and follow-up), we found a non-significant BCVA decrease compared with controls (baseline and follow-up), and none of the patients had complained of visual symptoms. 

In ALS patients compared with controls (baseline and follow-up in both groups), we did not find significant differences in the VF parameters, except in the percentage of fixation loss. We think that this difference could be due to two factors: Patients who were experiencing more neurological disability did not participate in the second exploration, thus there were fewer patients in the ALS-follow-up (*n* = 10) than in ALS-baseline (*n* = 20) group. As no studies analyzing VFs in ALS patients were available, we applied the criteria used to conduct VF in Parkinson disease (PD) patients [49]. The ALS patients have worse reliability indicators, such as a higher rate of fixation losses, as well as false positives and negatives, than the control group. In our study, ALS patients experienced difficulties, especially with handwriting and salivation in the ALSFRS-R. Both problems could affect the execution of the VF test due to the difficulty in maintaining the correct posture and pushing the button of the campimeter. Thus, it appears that the VF would not be an adequate test to evaluate ALS patients. 

In the OCT analysis, we found retinal thickness changes in ALS. The MT in temporal and inferior IMR areas was significantly thickened in the ALS baseline group compared with the control baseline group. Other authors have detected non-significant increased thickness in the retina of these patients, especially in the outer plexiform layer (OPL) [7,40,41]. This macular increased thickness has also been found in other neurodegenerative diseases such as early Alzheimer disease (AD) [34] or Down’s syndrome [31]. We hypothesize that the significant increase in MT in temporal and inferior IMR areas observed in our ALS patients could be caused by a process of protein aggregation [42,52] and/or neuroinflammation, in which microglia have an important role in ALS pathogenesis [56]. Activated microglia exhibit morphological changes (retraction of processes and enlargement of the soma), migrate, and proliferate. Thus, microglia could be the cause of the increased thickness observed in some retinal areas in our study. Microglial activation has been reported in AD and PD patients in the brain and retina, and only in the brain in ALS patients in relation to protein aggregates and degenerated neurons [42,52,57,58,59,60]. Ringer et al. analyzed microglia in retinal sections in a mice ALS experimental model SOD1G93A, and did not detect these changes [61]. On the other hand, in a recent study, in a mouse model of ALS (lacking Ranbp2 in motorneurons and retinal ganglion cells) in retinal whole-mounts, microglial activation was demonstrated [62]. Discrepancies between both studies can be due to the different methods used to study the retinal microglia. Ringer et al.’s [61] study used retinal sections whereas Cho et al.’s [62] study evaluated the microglial in retinal whole-mounts. The whole-mounts can allow a more reliable study of the microglial activation signs because microglial cells can be observed entirely. In addition, the increased thickness observed in our study could also be due to alterations in axonal transport. It has been proposed that axonal transport defects constitute one early molecular pathological mechanism in ALS mouse models [63]. Moreover, motorneurons rely particularly on mitochondria to buffer calcium and reduce the numbers of axonal mitochondrial due to the impairment of axonal transport and the disruption of calcium homeostasis. These changes reduce microtubule stability, worsening the bind of motor proteins to the microtubules, and thus, axonal transport enters in a vicious circle [64]. These observations suggest that defects in axonal transport could be responsible for the increase in soma size and the axonal calibre of retinal ganglion cells, which could produce the increased thickness observed by OCT in our study.

Roth et al. [7] did not find significant alterations in the thickness of the optic nerve in ALS patients compared with a control group using OCT. However, in our study comparing ALS baseline vs. ALS follow-up, we found significant thinning, both in MT (inferior IMR and OMR areas) and pRNFL (superior and inferior quadrants and in sectors H3, H5, H6, and H12). Other studies using OCT have also demonstrated a thinning of the MT and pRNFL in these patients [40,41,42,43,44,45]. Volpe et al. [42] reported that in ALS patients compared with controls, the total macular volume was significantly thinner and 37.5% of ALS patients had a pRNFL average below the first percentile. They concluded that the total MT and pRNFL thickness were inversely correlated with the duration of the disease. In that study, the histopathological analysis showed a loss of retinal ganglion cell axons, which could explain the macular thinning observed using OCT [42]. Finally, Rohani et al. [45] suggested that the thinning of the pRNFL could be related to Wallerian degeneration secondary to the death of cortical neurons. Some studies [62] [16] showed a functional deficit in the sensory processing of the visual pathway to the brain measured by visual evoked potentials (VEPs), both in ALS patients and animal models. Cho et al. [62] found a delay in the latency of VEPs in an ALS animal model (lacking Ranbp2 in motorneurons and retinal ganglion cells), before changes of motor behavior occurred. This fact supports a delay in the transmission from the optic nerve to the visual cortex. Accordingly, Munte et al. [16] found electrophysiological evidence of a cortical involvement including visual areas in ALS patients.

Our results suggest that the MT increases in the temporal and inferior IMR sectors could be the first change found in the retina at the beginning of the disease. These results agree with the observations found in the early stages of AD, where macular increased thickness was also found [34]. In the longitudinal study (ALS baseline vs. ALS follow-up), the evolution from an increased thickness to a thinning, both in MT and pRNFL thickness, is remarkable. These changes in the thickness of both the macula and the pRNFL in the progression of ALS could explain why there are no significant differences between the baseline and follow-up ALS, since there could be a transition from thickening to thinning with an intermediate stage of normal thickness in the retina.

Differences found between our study and others in OCT measurements [7,40,41,42,43,45,52] could be due to the early stages of ALS in our patients. The duration of the disease in our patients (10.80 ± 5.5 months, ranging from 1 to 18 months) was shorter than in other published studies, including Roth et al. (42 ± 34 months, ranging from 7 to 166 months) [7], Ringelstein et al. (22.3 ± 22.5 months, ranging from 3 to 120 months) [40], Hübers et al. (ranging from 2 to 98 months) [41], Simonett et al. (43.2 ± 43.4 months, ranging from 10 to 197 months) [43], and Rohani et al. (14.5 ± 11.3 months) [45]. Therefore, classifying these patients according to the duration of the disease is important to obtain a better knowledge of the disease.

Discrepancies among different studies could also be due to: (1) The low number of participant patients, mainly because most of them do not complain of visual problems, and (2) ALS is a heterogeneous disease [41]. However, despite the lack or the low prevalence of visual problems, most studies concluded that these patients underwent retinal changes [40,41,42,43,44,45,52,53]. 

In the CNS, an asymmetric involvement in ALS has been suggested [65,66,67,68]. Rohani et al. [45] noticed that a reduction in the pRNFL thickness only present in the LE in ALS patients, especially in the superior and nasal quadrants. Our observations were similar. In the ALS baseline group, LE thicknesses were thinner than those in the RE, both in the macular and pRNFL areas, being statistically significant in the inferior-nasal quadrant of GCC and in sectors H7 and H9 of the pRNFL sectors. In the ALS follow-up group, pRNFL thickness was thinner in the LE in the temporal quadrant and in sectors H8 and H9.

Some studies using OCT in ALS patients did not find a correlation between OCT parameters and ALSFRS-R score [7,40,41,42,44]. However, Simonett et al. [43] found a correlation between the thinning of the macular area and worsening pulmonary function tests. Rohani et al. [45] found a direct correlation between ALSFRS-R score and average pRNFL thickness in most quadrants. In our study, the ALS baseline group showed a moderate correlation between OCT pRNFL parameters and the ALSFRS-R score. We found an inverse correlation in the inferior quadrant and sectors H5 and H6, and a direct correlation in sector H8. Notably, in these quadrants and sectors, significant pRNFL thickness changes were also found in the comparison of ALS baseline with ALS follow-up (Table 4 and Figure 5).

One of the most important contributions of this work was the follow-up study. To the best of our knowledge, this is the first ophthalmological longitudinal study in ALS patients. The scarcity of follow-up studies could be due to the difficulty of following up with ALS patients due to their neurological disability. One of the main limitations of our study is the low number of patients because of the restrictive inclusion criteria. Another limitation is the low number of patients who could attend the second exploration. Therefore, further longitudinal studies with more patients are needed. 

## 5. Conclusions

In conclusion, in ALS patients, OCT was the only ophthalmologic exploratory method that showed the first significant changes in the anterior visual system. The analysis of the OCT showed: (1) In ALS baseline vs. control baseline, a significant increased macular thickness in the temporal and the inferior areas of the inner macular ring; (2) in ALS follow-up vs. ALS baseline, a significant macular thinning in the inferior areas of the inner and outer macular ring; (3) in ALS follow-up vs. ALS baseline, a pRNFL significant thinning in the superior and inferior quadrants and H3, H5, H6, and H12; and (4) ALS patients showed a moderate correlation between some OCT pRNFL parameters and the ALSFRS-R score. The retina is a window to the central nervous system. Thus, OCT could be used as a complementary and clinical tool for the diagnosis and prognosis of ALS (and in particular, of fast progressing ALS). 

## Figures and Tables

**Table 1:** Demographic data, Amyotrophic Lateral Sclerosis Functional Rating Scale-Revised, and visual field.

	Control		ALS		*p*-Value				Spearman Correlation ALSFRS-R	*p*-Value Spearman Correlation
	Baseline	Follow-Up	Baseline	Follow-Up	Control Baseline vs. Control Follow-Up	Control Baseline vs. ALS Baseline	Control Follow-Ip vs. ALS Follow-Up	ALS Baseline vs. ALS Follow-Up		
	*n* = 38	*n* = 38	*n* = 20	*n* = 10						
Gender (Male/Female)	9/10	7/1	12/8	4/6						
	Mean ± SD									
BCVA **	0.98 ± 0.06	0.98 ± 0.04	0.90 ± 0.16	0.91 ± 0.13	0.487	0.230	0.146	0.354	0.317	0.173
**AGE**	44.74 ± 11.81	45.54 ± 12.47	51.10 ± 9.89	51.30 ±10.02	1.000	**0.044**	0.250	0.157	(−0.156)	0.338
IOP **	15.08 ± 2.22	15.31 ± 1.89	15.75 ± 1.99	15.20 ± 1.48	0.146	0.365	0.979	0.564	(−0.407)	0.075
ALSFRS-R **	48.00 ± 0.00	48.00 ± 0.00	29.50 ± 14.89	35.6 ± 14.08	1.000	<**0.001**	<**0.001**	0.068	------	------
VFI **	98.63 ± 1.55	99.13 ± 1.09	95.00 ± 8.51	94.63 ± 10.08	0.138	0.388	0.084	0.999	(−0.047)	0.852
MD **	(−0.76) ± 1.68	(−0.05) ± 1.09	(−1.77) ± 3.74	(−2.66) ± 4.71	0.161	0.902	0.086	0.148	0.0854	0.736
PSD **	1.76 ± 0.75	1.68 ± 0.62	2.68 ± 2.20	2.87 ± 2.96	0.877	0.297	0.257	0.896	(−0.058)	0.818
% FL **	22.87 ± 28.99	28.23 ± 31.92	28.12 ± 35.06	10.21 ± 12.56	0.615	0.679	0.150	**0.037**	(−0.052)	0.839
% FN **	1.45 ± 2.80	2.19 ± 3.04	3.06 ± 4.02	3.5 ± 3.29	0.143	0.407	0.315	0.609	(0.152)	0.546
% FP **	2.68 ± 3.50	3.25 ± 4.07	3.44 ± 4.19	1.5 ± 2.27	0.850	0.643	0.287	0.165	(−0.131)	0.605

** Non parametric. Data are expressed as mean ± standard deviation (SD) (except sex). Numbers in bold denote *p* < 0.05 after Bonferroni correction. BCVA: Best-corrected visual acuity; IOP: Intraocular pressure; ALS: Amyotrophic lateral sclerosis; ALSFRS-R: Amyotrophic Lateral Sclerosis Functional Rating Scale-Revised. Parameters that measure retinal sensitivity: VFI: Visual field index; MD: Mean deviation (dB); PSD: Pattern standard deviation. Parameters that measure visual field reliability: % FL: Percentage of fixation losses; % FN: Percentage of false negative errors; % FP: Percentage of false positive errors.

**Table 2:** Analysis of macular thickness measures by OCT between ALS and control group.

		Control		ALS		*p*-Value				Spearman Correlation ALSFRS-R	*p*-Value Spearman Correlation
		Baseline	Follow-Up	Baseline	Follow-Up	Control Baseline vs. Control Follow-Up	Control Baseline vs. ALS Baseline	Control Follow-Up vs. ALS Follow-up	ALS Baseline vs. ALS Follow-Up		
Macular Analysis (µm)		*n* = 38	*n* = 38	*n* = 20	*n* = 10						
		Mean ± SD									
**Fovea o CMT**		261.42 ± 21.31	255.81 ± 23.61	262.10 ± 26.29	274.90 ± 25.32	0.244	1.000	0.567	0.335	0.275	0.241
**IMR**	**Superior**	320.55 ± 12.30	315.31 ± 12.06	327.55 ± 15.31	322.20 ± 12.63	0.526	0.435	1.000	0.166	0.077	0.746
	**Temporal**	307.32 ± 12.49	303.06 ± 14.21	315.85 ± 15.34	313.40 ± 13.38	0.705	**0.049**	1.000	0.160	0.079	0.741
	**Nasal**	323.40 ± 13.99	319.13 ± 15.15	330.35 ± 16.00	327.40 ± 10.93	0.794	0.510	1.000	0.397	0.159	0.502
	**Inferior**	319.00 ± 12.54	314.88 ± 14.58	326.91 ± 15.34	320.00 ± 11.88	0.660	**0.029**	1.000	**0.002**	0.000	0.852
**OMR**	**Superior**	280.30 ± 10.95	279.06 ± 11.38	279.65 ± 14.39	271.00 ± 14.73	0.444	1.000	0.418	0.161	(−0.121)	0.613
	**Temporal**	260.05 ± 10.04	256.63 ± 6.42	264.58 ± 13.90	261.20 ± 12.54	0.937	0.912	0.872	0.327	(−0.160)	0.498
	**Nasal**	298.97 ± 13.46	295.38 ± 14.39	297.49 ± 24.05	292.20 ± 17.51	0.649	1.000	1.000	0.652	0.057	0.812
	Inferior **	269.74 ± 12.84	269.31 ± 9.44	274.42 ± 14.20	264.40 ± 16.34	0.183	0.819	1.000	**0.031**	(−0.230)	0.329
**Cube Volume**		10.08 ± 0.41	10.04 ± 0.29	10.14 ± 0.49	10.00 ± 0.41	0.309	1.000	0.292	0.120	(−0.104)	0.661
**Cube average thickness**		279.58 ± 11.23	279.06 ± 7.83	281.70 ± 13.79	277.70 ± 11.70	0.188	1.000	0.181	0.135	(−0.100)	0.674

** No parametric. Data are expressed as mean ± SD. Numbers in bold indicate *p* < 0.05 after Bonferroni correction. IMR: Inner macular ring; OMR: Outer macular ring; CMT: Central macular thickness.

**Table 3:** Analysis of ganglion cell complex measures by OCT between ALS and control group.

GCC Analysis (µm)		Control		ALS		*p*-Value				Spearman Correlation ALSFRS-R	*p*-Value Spearman Correlation
		Baseline	Follow-Up	Baseline	Follow-Up	Control Baseline vs. Control Follow-Up	Control Baseline vs. ALS Baseline	Control Follow-Up vs. ALS Follow-Up	ALS Baseline vs. ALS Follow-Up		
		*n* = 38	*n* = 38	*n* = 20	*n* = 10						
		Mean ± SD									
**Superior**	Central **	81.63 ± 6.18	80.88 ± 7.62	78.70 ± 13.12	76.10 ± 10.04	0.999	1.000	1.000	0.805	(−0.179)	0.450
	Temporal **	79.08 ± 8.09	78.94 ± 4.92	77.80 ± 9.36	76.00 ± 10.03	0.588	1.000	1.000	0.601	0.020	0.934
	**Nasal**	82.68 ± 6.98	82.13 ± 5.81	80.10 ± 10.80	77.50 ± 9.99	0.458	1.000	1.000	0.957	0.103	0.667
**Inferior**	**Central**	80.53 ± 6.90	80.75 ± 4.67	80.35 ± 9.69	73.70 ± 12.15	0.989	1.000	0.407	0.070	(−0.237)	0.314
	**Temporal**	81.40 ± 5.53	80.25 ± 4.68	81.00 ± 10.05	77.30 ± 9.81	0.327	1.000	1.000	0.212	(−0.196)	0.407
	**Nasal**	81.32 ± 7.65	80.50 ± 4.90	77.20 ± 11.28	75.20 ± 12.21	0.873	1.000	0.872	0.284	0.085	0.721
**GCL (µm) thickness**	**Average**	81.18 ± 6.20	80.56 ± 5.20	79.15 ± 9.50	76.00 ± 10.30	0.592	1.000	1.000	0.336	(−0.011)	0.962
	Minimum **	78.90 ± 6.13	78.06 ± 5.70	72.70 ± 15.04	74.00 ± 11.74	0.184	1.000	1.000	0.347	0.389	0.090

** No parametric. Data are expressed as mean ± SD. Numbers in bold indicate *p* < 0.05 after Bonferroni correction. ALS: Amyotrophic lateral sclerosis; OCT: Optical coherence tomography; GCC: Ganglion cell complex; GCL: Ganglion cell layer; µm: Microns.

**Table 4:** Analysis of peripapillary retinal nerve-fiber layer measures by OCT between ALS and control group.

Peripapillary Analysis (µm)		Control		ALS		*p*-Value				Spearman Correlation ALSFRS-R	*p*-Value Spearman Correlation
		Baseline	Follow-Up	Baseline	Follow-Up	Control Baseline vs. Control Follow-Up	Control Baseline vs. ALS Baseline	Control Follow-Up vs. ALS Follow-Up	ALS Baseline vs. ALS Follow-Up		
		*n* = 38	*n* = 38	*n* = 20	*n* = 10						
		Mean ± SD									
**Average RNFL Thickness**		94.84 ± 12.88	95.69 ± 13.82	88.00 ± 2.60	79.90 ± 13.75	0.572	0.659	1.000	0.073	(−0.334)	0.150
**Peripapillary RNFL Quadrants (µm)**	**Superior**	112.90 ± 18.33	117.38 ± 21.54	104.80 ± 23.80	93.50 ± 24.38	0.578	0.921	0.093	**0.049**	(−0.140)	0.555
	**Temporal**	64.95 ± 10.09	65.88 ± 7.21	64.35 ± 12.64	63.50 ± 15.81	0.935	1.000	0.742	0.100	0.362	0.117
	**Inferior**	130.85 ± 22.27	129.75 ± 24.70	116.65 ± 25.08	101.20 ± 26.62	0.074	0.447	1.000	**0.001**	(−0.527)	**0.017**
	**Nasal**	71.55 ± 14.87	69.94 ± 12.92	68.75 ± 17.42	61.30 ± 12.29	0.986	1.000	0.659	0.060	(−0.062)	0.795
**Peripapillary RNFL Sectors (µm)**	**H1 RE/H11 LE**	100.87 ± 20.30	100.94 ± 26.74	90.40 ± 22.74	84.30 ± 29.35	0.070	0.567	1.000	0.781	(−0.056)	0.814
	**H2 RE/H10 LE**	90.11 ± 25.28	92.63 ± 25.95	79.65 ± 23.86	75.10 ± 21.01	0.864	0.645	0.962	0.157	0.117	0.624
	**H3 RE/H9 LE**	56.76 ± 10.91	55.75 ± 6.59	60.30 ± 13.82	51.90 ± 10.40	0.452	1.000	0.330	**0.034**	(−0.318)	0.172
	**H4 RE/H8 LE**	63.82 ± 13.33	61.75 ± 10.99	65.55 ± 20.61	56.90 ± 12.88	0.566	1.000	1.000	0.320	(−0.019)	0.937
	**H5 RE/H7 LE**	111.37 ± 30.21	110.38 ± 22.80	102.40 ± 35.00	80.30 ± 17.93	0.635	1.000	0.126	**0.038**	(−0.647)	**0.002**
	**H6 OU**	148.16 ± 32.02	149.19 ± 30.94	122.75 ± 37.06	99.00 ± 31.24	0.091	0.109	0.510	**<0.001**	(−0.521)	**0.018**
	**H7 RE/H5 LE**	133.42 ± 26.05	129.50 ± 34.27	124.50 ± 30.54	123.50 ± 36.77	0.282	1.000	1.000	0.624	0.101	0.673
	**H8 RE/H4 LE**	65.13 ± 12.86	65.13 ± 11.04	67.35 ± 18.23	69.00 ± 18.74	0.837	1.000	1.000	0.024	0.488	0.029
	**H9 RE/H3 LE**	51.18 ± 8.79	53.38 ± 8.80	50.45 ± 9.59	47.50 ± 10.47	0.369	1.000	0.366	0.286	(−0.009)	0.970
	**H10 RE/H2 LE**	78.45 ± 16.84	79.19 ± 17.05	75.20 ± 16.95	74.00 ± 21.39	0.450	1.000	1.000	0.538	0.029	0.904
	**H11 RE/H1 LE**	129.92 ± 33.30	125.50 ± 48.73	117.25 ± 25.27	114.70 ± 29.59	0.307	0.629	0.924	0.747	(−0.098)	0.680
	**H12 OU**	108.63 ± 23.70	119.13 ± 20.47	101.50 ± 31.37	81.20 ± 26.15	0.350	1.000	0.060	**0.013**	(−0.335)	0.149

Data are expressed as mean ± SD. Numbers in bold denote *p* < 0.05 after Bonferroni correction. ALS: Amyotrophic lateral sclerosis. H: Clock-hour position; RNFL: Retinal nerve-fiber layer; C/D: Cup-to-disc.
